# Seroprevalence of Human T-Cell Lymphotropic Virus–1 in a Jamaican Antenatal Population and Assessment of Pooled Testing as a Cost Reduction Strategy for Implementation of Routine Antenatal Screening

**DOI:** 10.4269/ajtmh.23-0118

**Published:** 2023-10-23

**Authors:** Ynolde E. Leys, Jenene Cameron, Velesha Frater, Kaesha Thomas, Tiffany R. Butterfield, Michelle Campbell Mitchell, Cathy Maddan, Jacynth Moore, Russell Pierre, Gavin A. Cloherty, Joshua J. Anzinger

**Affiliations:** ^1^ Department of Microbiology, The University of the West Indies, Kingston, Jamaica, West Indies;; ^2^ Department of Obstetrics and Gynaecology, The University of the West Indies, Kingston, Jamaica, West Indies;; ^3^ Department of Child and Adolescent Health, The University of the West Indies, Kingston, Jamaica, West Indies;; ^4^ Infectious Diseases Research, Abbott Laboratories, Abbott Park, Illinois;; ^5^ Abbott Pandemic Defense Coalition, Abbott Park, Illinois;; ^6^ Abbott Pandemic Defense Coalition, The University of the West Indies, Kingston, Jamaica, West Indies;; ^7^ Global Virus Network, Baltimore, Maryland

## Abstract

Mother to child transmission (MTCT) of human T-cell lymphotropic virus (HTLV)–1 is associated with increased risk of adult T-cell leukemia and can be unrecognized without routine antenatal screening. We assessed the seroprevalence of HTLV-1/2 among pregnant women attending The University Hospital of the West Indies Antenatal Clinic, 2019, and validated a cost-effective strategy to screen antenatal clinic attendees for HTLV-1/2. Residual antenatal samples from 370 women were tested for HTLV-1/2 by chemiluminescence microparticle immunoassay (CMIA). Six samples were confirmed HTLV-1 positive by Western blot (none for HTLV-2) for a prevalence of 1.62%. Four mother–child pairs were able to be recruited for HTLV testing of children, with two children testing HTLV-1/2 positive. Medical records of HTLV-1–infected women revealed that all women breastfed, indicating an unrecognized risk for HTLV MTCT. To assess whether pooling of samples as a cost-reduction strategy could be introduced, we pooled all antenatal samples received between November and December 2021 into 12 pools of eight samples/pool. Two pools were CMIA positive, and de-pooling of samples identified two CMIA-positive samples (one per pool), both confirmed as HTLV-1 by Western blot. These results indicate that HTLV-1 remains prevalent in pregnant Jamaican women and that sample pooling can be a cost-effective strategy to limit MTCT in Jamaica.

## INTRODUCTION

Infection with human T-cell lymphotropic virus (HTLV) is lifelong and can lead to poor quality of life and substantially reduced life span if malignancy develops. Human T-cell lymphotropic virus–1 is associated with an aggressive malignancy and adult T-cell leukemia (ATL), as well as an inflammatory disease of the central nervous system known as HTLV-associated myelopathy or tropical spastic paraparesis (HAM/TSP).^1^ Other HTLV types include HTLV-2,^2^ HTLV-3,^3^ and HTLV-4,^4^^,^^5^ though all are much less common than HTLV-1. Human T-cell lymphotropic virus–1 is the only type associated with oncogenic properties and is the most common type to be associated with disease^6^; however, there remains no cure for HTLV-1, and treatment of the disease remains costly and the outcome is poor. For example, ATL, a universally fatal disease, has been shown in the United States to incur annual hospital expenses > U.S. $100,000 per patient per year.^7^ Pharmacotherapy such as integrase strand-transfer inhibitors and nucleoside analogue reverse-transcriptase inhibitors has demonstrated in vitro activity against HTLV-1; however, it has yet to show activity in vivo. In a pilot study, raltegravir was administered for 6 months to HTLV-1–infected persons, but it showed little reduction in HTLV-1 proviral load.^8^

Shortly after the initial discovery of HTLV-1 in 1980, HTLV-1 research flourished; however, the number of published HTLV-1 studies has declined since just before the 21st century. This limited research has resulted in a lack of basic knowledge, such as unclear estimates of the burden,^9^ and has led some experts to consider HTLV-1 a neglected disease.^9^^,^^10^ The current estimate of individuals infected with HTLV-1 is approximately 5 to 10 million people; however, these estimates are based solely on data collected from known endemic areas where epidemiological data are available, accounting for around 1.5 billion individuals. It is challenging to estimate the prevalence for densely populated regions such as India and China, and it is therefore likely that the actual number of HTLV-1 carriers is significantly higher.^11^ The virus is highly prevalent in some countries, such as the southern part of the Japanese archipelago, large regions of sub-Saharan Africa, several areas in the Caribbean and South America, Iran, and some areas of Australo-Melanasia.^12^ Most of these are developing countries that may not have adequate testing to identify infected persons and limit onward transmission. Furthermore, the lack of routine surveillance can lead to unclear estimates of prevalence. In Jamaica, after the initial epidemiological descriptions of HTLV, research has largely focused on HTLV-associated disease. An assessment of the current HTLV prevalence and strategies to reduce transmission in Jamaica are lacking.

The routes of HTLV-1 transmission include sexual transmission and transfusion of blood and blood products, as well as mother to child transmission (MTCT) primarily through breastfeeding and, rarely, zoonotic transmission of simian T-cell lymphotropic virus-1, which occurs mainly through severe nonhuman primate bites.^12^ Mother to child transmission is one of the most important transmission routes, as it is associated with the greatest risk of developing ATL^13^ and is also associated with increased risk of infective dermatitis and HAM/TSP. In late 20th century Jamaica (1983–1985), the prevalence of HTLV-1 infection in pregnant women as reported to be one of the highest in the world, with a seroprevalence as high as 3.5%,^14^ and a 2% (95% CI: 0.6–5.0%) seroprevalence was reported in 1988 for the same antenatal clinic.^15^ A knowledge attitudes and practices study of Jamaican women participating in the Jamaica Breastfeeding Intervention Study indicated limited knowledge of HTLV disease and transmission among study participants and showed that the majority of women were multiparous,^16^ indicating risk for HTLV MTCT to multiple children.

Prevention strategies to reduce transmission of HTLV have been implemented in several countries, including Japan, which implemented nationwide screening of all pregnant women in 2011.^17^ Mother to child transmission prevention methods for HTLV-infected pregnant women vary by prefecture but may include exclusive formula feeding, short-term breastfeeding, or freeze-thawed breastmilk feeding.^18^ Key to implementing these prevention strategies is identifying HTLV-infected pregnant women. However, in a low- and middle-income setting, the expense of testing may be a substantial obstacle, and strategies to limit cost may be essential.

In 2021, the WHO published a technical report on HTLV-1 highlighting the need for prevalence data and further studies on transmission, especially MTCT.^8^ In this study, we sought to determine the prevalence of HTLV infection in pregnant Jamaican women attending an antenatal clinic and to identify a cost-reduction strategy for implementation of routine HTLV testing.

## MATERIALS AND METHODS

### Study design.

Residual serum samples from antenatal patients attending the University Hospital of the West Indies antenatal clinic in 2019 were used for this study. A total of 926 unique antenatal patient serum samples were submitted for routine antenatal virology screening in 2019. To obtain a 95% CI with a 4% margin of error, 370 samples (40% of the total) were selected for HTLV-1/2 antibody testing. Forty percent of antenatal samples for each month of 2019 were selected randomly to ensure representative sampling between months. Sample testing was performed in 2020.

### Laboratory tests.

Human T-cell lymphotropic virus–1/2 antibody testing was performed using the Abbott ARCHITECT *r*HTLV-I/II assay (Abbott GmbH & Co., Wiesbaden, Germany), which qualitatively detects antibodies to HTLV-1 and HTLV-2 in human serum or plasma by the chemiluminescent microparticle immunoassay (CMIA) method. Samples were tested according to the manufacturer’s instructions with a signal/cutoff (S/CO) value of ≥ 1 considered HTLV-1/2 reactive. Reactive serum samples were retested, and residual serum from the source tube clot was also tested to confirm reactivity; repeatedly reactive samples were tested by Western blot (MP Diagnostics HTLV Blot 2.4, Solon, Ohio) to confirm CMIA results and differentiate between HTLV-1 and HTLV-2. Western blot interpretation was according to the manufacturer’s instructions. The managing physician was informed of patients with confirmed HTLV infections. Women were notified of the result, with some opting to return to the clinic to have their child tested for HTLV using the same testing strategy described above. The children were > 1 year old, and therefore no maternal antibodies would be expected to be present.^19^ Clinical data for HTLV-positive antenatal mothers were collected from the patient’s medical docket.

### Pooling of serum samples.

To determine a pool size in which all samples testing positive individually would also test positive when pooled, samples with the lowest and the highest S/CO by CMIA were serially diluted with HTLV-1/2 negative (CMIA) sera and tested with the Abbott ARCHITECT *r*HTLV-I/II assay.

To validate the pooling method, all (96) antenatal clinic samples received from November 1 to December 12, 2021 were combined in 12 pools of eight samples per pool and tested by CMIA. Reactive pools were de-pooled, and specimens were retested individually. Samples that were reactive by CMIA were tested by Western blot (MP Diagnostics HTLV Blot 2.4) for confirmation.

### HTLV test cost analysis.

Costs in Jamaican dollars were converted into U.S. dollars using the average monthly exchange rate per the Bank of Jamaica.^20^ Shipping and handling were excluded from the cost analysis but were expected to be similar for individual and pooled testing. Cost analysis was determined for quarterly testing and based on 926 samples received in 2019 and pooling of eight samples for pooled testing. Individual testing included 926 samples tests and 8 control tests (2 per quarter); pooled testing included 116 pool tests, 184 de-pooled tests (from anticipated 23 HTLV-positive pools), and 8 control tests (2 per quarter). Abbott ARCHITECT *r*HTLV-I/II assay reagents have a shelf life of 6 months; therefore, calibration was required twice for a year of testing.

### Statistical analysis.

Demographic data were compared between HTLV-1–infected and –uninfected women with a Student’s *t* test for age and χ^2^ test for all other data. A *P* value < 0.05 was considered statistically significant. Clopper-Pearson confidence intervals were calculated for prevalence estimates.

## RESULTS

### HTLV seroprevalence of an antenatal clinic.

Human T-cell lymphotropic virus–1/2 seroprevalence of antenatal clinic attendees for calendar year 2019 was determined by assessing HTLV-1/2 reactivity by CMIA and subsequent Western blot testing of all positive samples for confirmation (Table 1). Of the 370 samples tested by CMIA, nine samples were repeatedly HTLV-1/2 reactive. There were no reactive samples with discordant confirmatory repeat CMIA test results. Six of the nine samples that were reactive for HTLV-1/2 had high S/CO values (> 90 S/CO), and three samples had low S/CO values (< 7 S/CO). Western blot testing (Supplemental Figure 1) showed that all six samples testing high positive (> 90 S/CO) by CMIA as HTLV-1/2 were confirmed HTLV-1 infections, two of three low positives by CMIA were determined to be false-positives, and the remaining low positive was indeterminate. Considering all CMIA low positives as HTLV negative, the prevalence of HTLV-1 was 1.6% (6/370; 95% CI: 0.6–3.5%). None of the HTLV-1/2 CMIA reactive samples tested HTLV-2 positive by Western blot (Supplemental Figure 1), and none were HIV positive via the Abbott ARCHITECT HIV-1/2 Ag/Ab Combo Assay (data not shown).

**Table 1:** Prevalence assessment results of antenatal samples identified as HTLV-1/2 CMIA reactive

Patient	CMIA result (S/CO)	Western blot result
1	90.17	HTLV-1 positive
2	115.63	HTLV-1 positive
3	141.31	HTLV-1 positive
4	3.29	HTLV-1/2 negative
5	177.31	HTLV-1 positive
6	111.52	HTLV-1 positive
7	4.04	HTLV-1/2 indeterminate
8	109.79	HTLV-1 positive
9	6.93	HTLV-1/2 negative

CMIA = chemiluminescent microparticle immunoassay; HTLV = human T-cell lymphotropic virus; S/CO = signal/cutoff.

Of the six women with confirmed HTLV-1 infections, four women and their children were able to be recruited for HTLV-1/2 testing. The ages of these children ranged from 14 to 21 months. Of these four children from HTLV-1–infected women (one child for each of the four mothers), two were HTLV-1/2 reactive (S/CO 144.71 and 16.92) by CMIA. Human T-cell lymphotropic virus–1 infection was confirmed for one child (21 months old; S/CO 144.71) by Western blot (Supplemental Figure 1), and the sample from the other child (18 months old) had insufficient volume for testing.

### Demographics of HTLV-infected and HTLV-uninfected women.

Having established that HTLV-1 infection in the antenatal population remains endemic in Jamaica, essentially unchanged from more than two decades earlier,^15^ together with evidence of MTCT, we next sought to determine if testing HTLV-1 positive was associated with any demographic data as a potential means for targeted testing of a group most likely to be HTLV-1 infected (Table 2). Clinical records were only available for all six HTLV-1–infected women and 244 HTLV-uninfected women (i.e., women testing CMIA positive and Western blot negative or indeterminate were excluded from the analysis). The age range of the women tested for HTLV was 17 to 46 years old and was similar for HTLV-1–infected and –uninfected women, with a mean (SD) age of 27.17 ± 6.66 years for HTLV-1–infected women and 28.24 ± 6.07 years for HTLV-1–uninfected women. All HTLV-1–infected women commenced breastfeeding immediately after delivery, but information regarding the duration of breastfeeding was not available. Three of the six women had previous miscarriages, two women had one spontaneous miscarriage each, and the other one had an induced miscarriage. When HTLV-1–infected and –uninfected women were compared, parity (not inclusive of current pregnancy), breastfeeding (children from the pregnancy examined for prevalence assessment), number of previous miscarriages, comorbidities, and education level completed were all similar. There was also no obvious difference in these characteristics between re-recruited HTLV-1–infected mothers with HTLV-1–infected children and re-recruited HTLV-1–infected mothers with HTLV-1–uninfected children (Supplemental Table 1).

**Table 2:** Demographic data of HTLV-1–positive and –negative women

Characteristic	Total* (*N* = 250)	HTLV positive (*n* = 6)	HTLV negative (*n* = 244)	*P* value
Mean age ± SD, years	28.22 ± 6.02	27.17 ± 6.66	28.24 ± 6.07	0.51
Pregnancy history				
Parity				
0–1	77.2%	100.0%	76.7%	0.46
≥ 2	22.8%	0.0%	23.3%	
Previous miscarriage				
0	59.7%	33.3%	60.3%	0.38
1	29.6%	66.7%	25.6%	
> 2	13.7%	0.0%	14.0%	
Pre-term delivery	11.9%	–	12.2%	0.79
Breastfeeding history				
At birth	79.6%	100.0%	79.2%	0.43
At birth unknown	20.3%	–	20.8%	–
At 6 weeks	76.9%	100.0%	76.3%	0.23
Comorbidities				
At least one comorbidity	28.3%	–	29.0%	–
Respiratory illness	17.1%	–	17.6%	–
Hypertension	4.8%	–	4.9%	–
Sickle cell	0.4%	–	0.4%	–
Diabetes mellitus	1.2%	–	1.2%	–
Autoimmune disease	0.8%	–	0.8%	–
Other†	5.2%	–	5.2%	–
Multimorbidity (2+)	10.3%	–	10.6%	–
Education				
Secondary	42.0%	16.7%	42.6%	0.51
Tertiary	52.8%	66.7%	52.5%	

HTLV = human T-cell lymphotropic virus.

* Western blot negative (two) and indeterminate (one) samples were not included in the analysis.

† Other chronic illnesses are neurological disorders, anemia, and cardiovascular disease.

### Assessment of pooled HTLV testing.

As demographic data were similar for HTLV-1–infected and –uninfected women, identifying groups most likely to be HTLV-1 infected was not possible, indicating the need for indiscriminate testing of all samples to identify HTLV-infected women. In the absence of a targeted testing approach to reduce testing costs, we next determined if pooled testing of samples could be a suitable strategy for routine antenatal HTLV testing. We first determined if pooling of antenatal samples would be possible to identify HTLV-infected women by CMIA. Signal/cutoff values were determined for a diluted high and low CMIA positive sample (Supplemental Figure 2). Results of 2-fold dilutions showed that the high positive sample remained positive at least up to a 128-fold dilution, whereas for the low positive, an 8-fold dilution remained positive but became negative when diluted 16-fold. Based on these dilution results, we then assessed the most cost-effective number of samples per pool to test all (96) antenatal samples received during 6 consecutive weeks from November to December 2021. As 2.43% (9/370) of samples tested HTLV-1/2 CMIA reactive for our prevalence assessment, we determined that two samples would likely test positive for 96 samples examined (i.e., 2.43% × 96 = 2.3). Thus, 24 pools at 4 samples/pool and 12 pools at 8 samples/pool would require 32 tests (24 pools tested plus de-pooling of 2 pools of 4) and 24 tests (8 pools tested plus de-pooling of 2 pools of 8), respectively.

We next validated pooled testing of all (96) antenatal samples received during 6 consecutive weeks from November to December 2021 using pools of eight samples/pool (Table 3). Of the 12 pools tested, as expected, two pools were reactive by CMIA. De-pooling of the two reactive pools identified one sample from each pool reactive by CMIA, with both samples confirmed as HTLV-1 infections by Western blot (Supplemental Figure 1). These data are consistent with the 1.6% prevalence we identified among 370 women tested, with 2.1% (2/96) of samples confirmed HTLV-1 positive by pooled testing.

**Table 3:** Assessment of pooled HTLV-1/2 testing of antenatal samples

Pool no.	Pool CMIA result (S/CO)	De-pool no.	De-pool CMIA result (S/CO)	Western blot result
1	85.21	Pool 1, Sample 1	0.06	–
2	0.12	Pool 1, Sample 2	0.07	–
3	0.14	Pool 1, Sample 3	0.1	–
4	0.11	Pool 1, Sample 4	0.1	–
5	0.15	Pool 1, Sample 5	107.66	Positive
6	0.11	Pool 1, Sample 6	0.11	–
7	0.20	Pool 1, Sample 7	0.07	–
8	0.12	Pool 1, Sample 8	0.11	–
9	152.98	Pool 9, Sample 1	193.84	Positive
10	0.11	Pool 9, Sample 2	0.07	–
11	0.10	Pool 9, Sample 3	0.1	–
12	0.11	Pool 9, Sample 4	0.06	–
–	–	Pool 9, Sample 5	0.11	–
–	–	Pool 9, Sample 6	0.13	–
–	–	Pool 9, Sample 7	0.17	–
–	–	Pool 9, Sample 8	0.1	–

CMIA = chemiluminescent microparticle immunoassay; HTLV = human T-cell lymphotropic virus; S/CO = signal/cutoff.

### Cost analysis of pooled HTLV testing.

The validation of pooled HTLV CMIA testing prompted us to perform a cost analysis comparing pooled and individual HTLV testing (Table 4). Reagent costs were assessed for quarterly testing of first-trimester samples for all antenatal samples received during 2019. Compared with monthly testing, quarterly testing is more economical (fewer CMIA and Western blot controls/calibration required) and would still allow for identification of HTLV-infected pregnant women prior to delivery (as samples would be from the first trimester), still allowing for a potential intervention to reduce MTCT risk. We assessed the pooled testing cost by determining the total number of reagents required to identify all samples expected to test positive by CMIA from 926 samples, which we determined to be 23 based on the 2.43% (9/370) of samples that tested CMIA positive for our analysis above. Costs for pooled testing included testing of 116 pooled samples (926 samples/8 sample per pool) and testing of 184 de-pooled samples (23 anticipated positive pools). The total cost for individual and pooled testing was U.S. $6,259.98 and U.S. $3,923.42, respectively, with further cost reduction achieved for pooling by increasing the cutoff value (Supplemental Table 2). If antenatal HTLV-1/2 testing was to be done at the same time as routine HTLV testing, dedicated controls and calibrators would not be needed, and therefore the cost for individual testing and pooled testing would be U.S. $5,375.58 and U.S. $3,039.02, respectively.

**Table 4:** Cost analysis of quarterly pooled HTLV testing

Reagent	Reagent cost	Individual testing reagent quantity required*	Individual testing reagent cost	Pooled testing reagent quantity required†	Pooled testing reagent cost
Pre-trigger solution	$128.87	1	$128.87	1	$128.87
Trigger solution	$112.64	1	$112.64	1	$112.64
Wash buffer	$50.87	3	$152.61	1	$50.87
Probe conditioner	$85.86	1	$85.86	1	$85.86
Reaction vessel	$0.09	980	$88.20	354	$31.86
Sample cup	$0.05	980	$49.00	354	$17.70
*r*HTLV-I/II reagent test	$3.43	980	$3,361.40	354	$1,214.22
*r*HTLV-I/II control	$291.19	2	$582.38	2	$582.38
*r*HTLV-I/II calibrator	$151.01	2	$302.02	2	$302.02
Western blot (36 tests)‡	$1,397.00	1	$1,397.00	1	$1,397.00
Total cost	–	–	$6,259.98	–	$3,923.42

HTLV = human T-cell lymphotropic virus.

* Individual testing includes 926 sample tests, 46 confirmatory retests (from anticipated 23 HTLV-positive samples), and 8 control tests (2 per quarter).

† Pooled testing includes 116 pooled tests, 184 de-pooled tests (from anticipated 23 HTLV-positive pools), 46 confirmatory retests (from anticipated 23 HTLV-positive samples), and 8 control tests (2 per quarter).

‡ The smallest Western blot kit size available is 36 tests.

## DISCUSSION

Pregnant Jamaican women continue to be an important population affected by HTLV-1 infection, with an HTLV-1 seroprevalence of 1.62%, comparable to the 2% seroprevalence identified > 20 years earlier for the same antenatal clinic.^15^ In addition to identifying that HTLV-1 remains endemic in pregnant Jamaican women, with a nearly unchanged prevalence from > 20 years earlier, we showed that silent MTCT is likely occurring in the absence of routine testing and that pooled testing can be an economically feasible approach that can be used as a means to reduce MTCT.

The identification of HTLV-1–infected pregnant women is concerning as Jamaica currently does not offer routine antenatal screening for HTLV; this poses a risk for silent MTCT, particularly via breastfeeding, which was identified to be universal for all HTLV-1–infected mothers in this study. All HTLV-1–infected women in this study were HIV negative, and thus breastfeeding would likely be encouraged, as is endorsed by “The Baby Friendly Hospital Initiative” established in 1991, an ongoing global campaign supporting health care systems and breastfeeding initiatives as well as facilitating health care workers to promote same (Ministry of Health and Wellness, Jamaica). In a 2016 Jamaican study, 33,218 babies were followed up until they were 3 months old, and 47.5% were exclusively breastfed (Planning Institute of Jamaica). In Multiple Indicator Cluster Survey-Jamaica (2011), it was noted that there was a national increase in exclusive breastfeeding rates for up to 6 months from 15.2% to 23.8%, and on average, Jamaican women exclusively breastfed for 3 weeks.^21^ These findings indicate that without maternal HTLV diagnostic testing, as in Jamaica, breastfeeding likely remains an important means of transmission. All HTLV-1–infected women in this study were unaware of their HTLV status and breastfed, with evidence of HTLV-1 MTCT for two of four mothers.

The total duration of breastfeeding of HTLV-1–positive mothers in this study was not available in medical records; however, at the mothers’ 6-week postnatal clinic visit all mothers were still exclusively breastfeeding. Studies have demonstrated that breastfeeding for 6 months or longer has a greater association with MTCT.^22^ Up to 25% of babies born to HTLV-1–infected mothers become infected if they were breastfed for more than 12 months, but only 5% if strictly formula fed (presumably, intrauterine transmission or transplacental infection accounted for these infections).^23^ In a previous Jamaican study, breastfeeding for longer durations beyond 12 months had an associated transmission rate of 32%, in comparison to that of 9% when mothers breastfed for shorter durations.^23^ Thus, if HTLV-1–infected women identified by routine screening choose to breastfeed after counseling, short-term breastfeeding should be advised to reduce MTCT risk.

Our data show that HTLV-1 is prevalent in the population examined and suggests that it may be the main circulating HTLV type in Jamaica. Human T-cell lymphotropic virus–2 is far less prevalent worldwide than HTLV-1,^24^ but it can be highly prevalent among indigenous populations in the Americas as well as intravenous drug users.^25^^,^^26^ We did not identify any women infected with HTLV-2 in this study. Notably, Jamaica does not have an indigenous population (decimated during colonization), and intravenous drug use is extremely rare,^27^ potentially explaining the lack of HTLV-2 identified in this study.

Testing of the HTLV-1/2 reactive samples by Western blot showed that the three CMIA low reactive samples (S/CO 3.29–6.93) were likely false-positives, although it remains possible that these women were early in the process of seroconversion.^28^ Previous studies show that false-positive results with the Abbott ARCHITECT *r*HTLV-I/II assay are typically associated with low S/CO values, similar to what we found in this study.^29^^–^^32^ The number of false-positives (3/370) is in keeping with a previous analysis showing the Abbott ARCHITECT *r*HTLV-I/II assay specificity of 98.1%.^33^ Our identification of false-positives reinforces the need for confirmatory testing to ensure misdiagnosis does not occur. Additionally, Western blot differentiates between HTLV-1 and -2, which allows for determination of disease burden, as these two different HTLV types have different disease burdens.

One approach to limiting testing due to economic constraints is identification of risk factors associated with HTLV-1 infection for targeted testing. However, in the population examined, we did not identify any demographic factors associated with testing HTLV-1 positive, indicating the need for HTLV testing of all women indiscriminately. Although this can potentially be cost-prohibitive in a low- and middle-income setting, we describe a quarterly pooled testing approach by CMIA that substantially reduces costs compared with individual testing. Pooling of sera has been used from 2002 to 2013 in the United Kingdom for blood bank screening, which reduced testing costs,^34^ and a study done in Brazil showed that pooling five samples/pool would result in a cost reduction of 60.7–73.6%.^35^ In a lower prevalence setting, costs could be further decreased with larger pools that could also negate the need for Western blot testing of CMIA low positive samples, in which most or all are false-positives,^29^ and would be expected to test CMIA negative with pools of 16 samples or greater; however, further studies in a low prevalence setting would be required to confirm this. Another approach that could be considered to further reduce the cost of HTLV screening in this population is increasing the cutoff value for HTLV reactive samples. As highlighted in this study and others,^29^^–^^32^ raising the cutoff value to ≥ 4 S/CO would result in a reduction in de-pooling tests (from the reduced number of pools testing positive) and confirmatory testing of these samples. Our data showed a false-positive sample with an S/CO of 6.93, so increasing the cutoff to ≥ 7 S/CO would have an even greater impact on cost reduction. Raising the test cutoff value is expected to result in some loss of sensitivity, which may result in a small number of persons testing false-negative. We are not aware of a previous study raising the test cutoff value for pooled testing. To ensure test performance is not negatively impacted, raising the test cutoff value for pooled testing should be validated. Finally, cost and sensitivity of a pooled testing strategy should be carefully considered and taken into the context of the setting.

The study was limited by its retrospective nature, in which we were unable to notify the managing physicians of results in real time to limit the possibility of vertical transmission and relied on recorded medical information that limited our ability to gain more information regarding child feeding practices of mothers. Because of the small number of mothers who were confirmed HTLV-1 positive and able to be re-recruited, it was not possible to obtain statistically meaningful comparisons between HTLV-1–positive mothers with an HTLV-positive child and those with an HTLV-negative child. We were also limited by the lack of peripheral blood mononuclear cell samples and polymerase chain reaction testing, which would have allowed for a more definitive diagnosis for the mother in this study who tested HTLV-1/2 CMIA positive but Western blot indeterminate.

The absence of HTLV screening and encouragement of breastfeeding is likely to facilitate the ongoing transmission of this virus in the Jamaican population. Jamaica is an upper middle-income economy with a high public debt and a poverty rate of 19% in 2017,^36^^,^^37^ limiting the ability to initiate high-cost testing programs. Our validated cost-effective strategy for HTLV screening could be implemented at our institute and, if successful, could be expanded to the public sector in which there were 34,637 births in 2019 (Statistical Institute of Jamaica). The approach of pool testing may not detect all HTLV-1/2–positive patients. For example, it is possible for women to become infected during pregnancy.^38^ Identification of HTLV-infected mothers at the national level could be a useful approach toward decreasing the prevalence, and hence disease, associated with HTLV infections in Jamaica.

## Financial Disclosure

This research was funded by a University of the West Indies Postgraduate Research Fund grant (Y. E. L.) and Abbott Laboratories. As Global Infectious Diseases Scholars, Y. E. L. and T. R. B. received mentored research training in the development of this manuscript. This training was supported in part by the University at Buffalo Clinical and Translational Science Institute award UL1TR001412 and the Global Infectious Diseases Research Training Program award D43TW010919. The content is solely the responsibility of the authors and does not necessarily represent the official views of the Clinical and Translational Science Institute or the National Institutes of Health.

## Supplemental files

10.4269/ajtmh.23-0118 Supplemental Materials
