# Supplementary material for: Seroprevalence of Human T-Cell Lymphotropic Virus–1 in a Jamaican Antenatal Population and Assessment of Pooled Testing as a Cost Reduction Strategy for Implementation of Routine Antenatal Screening
Source: Am J Trop Med Hyg. 2023 Oct 23;109(6):1344–50. doi: 10.4269/ajtmh.23-0118 (PMC10793066; doi:10.4269/ajtmh.23-0118)
Supplement: Supplemental Materials [file tpmd230118.SD1.pdf]

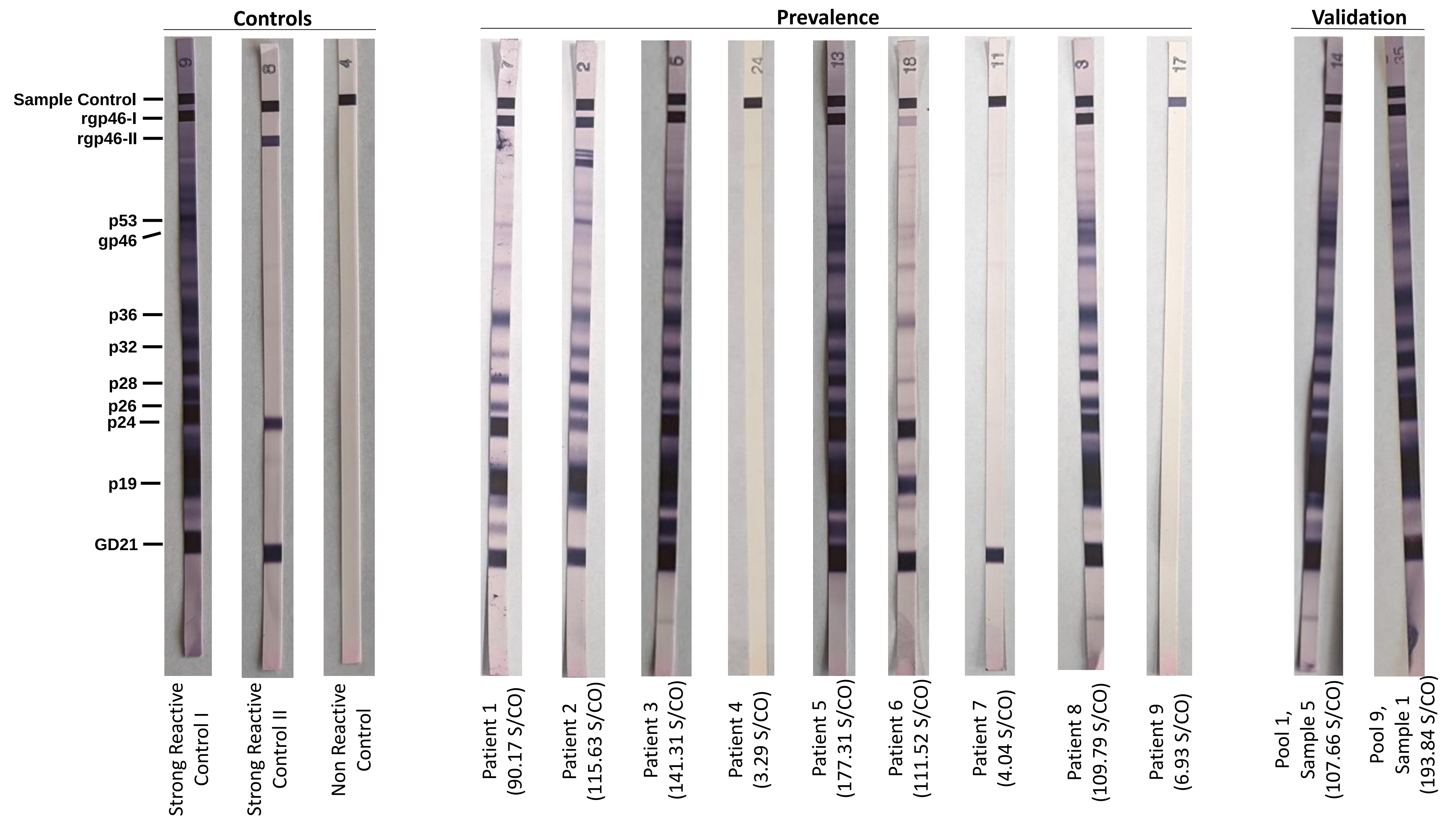

Controls
Prevalence
Validation
Sample Control
rgp46-I
rgp46-II
p53
gp46
p36
p32
p28
p26
p24
p19
GD21
Strong Reactive Control I
Strong Reactive Control II
Non Reactive Control
Patient 1 (90.17 S/CO)
Patient 2 (115.63 S/CO)
Patient 3 (141.31 S/CO)
Patient 4 (3.29 S/CO)
Patient 5 (177.31 S/CO)
Patient 6 (111.52 S/CO)
Patient 7 (4.04 S/CO)
Patient 8 (109.79 S/CO)
Patient 9 (6.93 S/CO)
Pool 1, Sample 5 (107.66 S/CO)
Pool 9, Sample 1 (193.84 S/CO)

Supplemental Figure 1. HTLV Western blots of HTLV-1/2 CMIA positive samples for prevalence and validation studies.

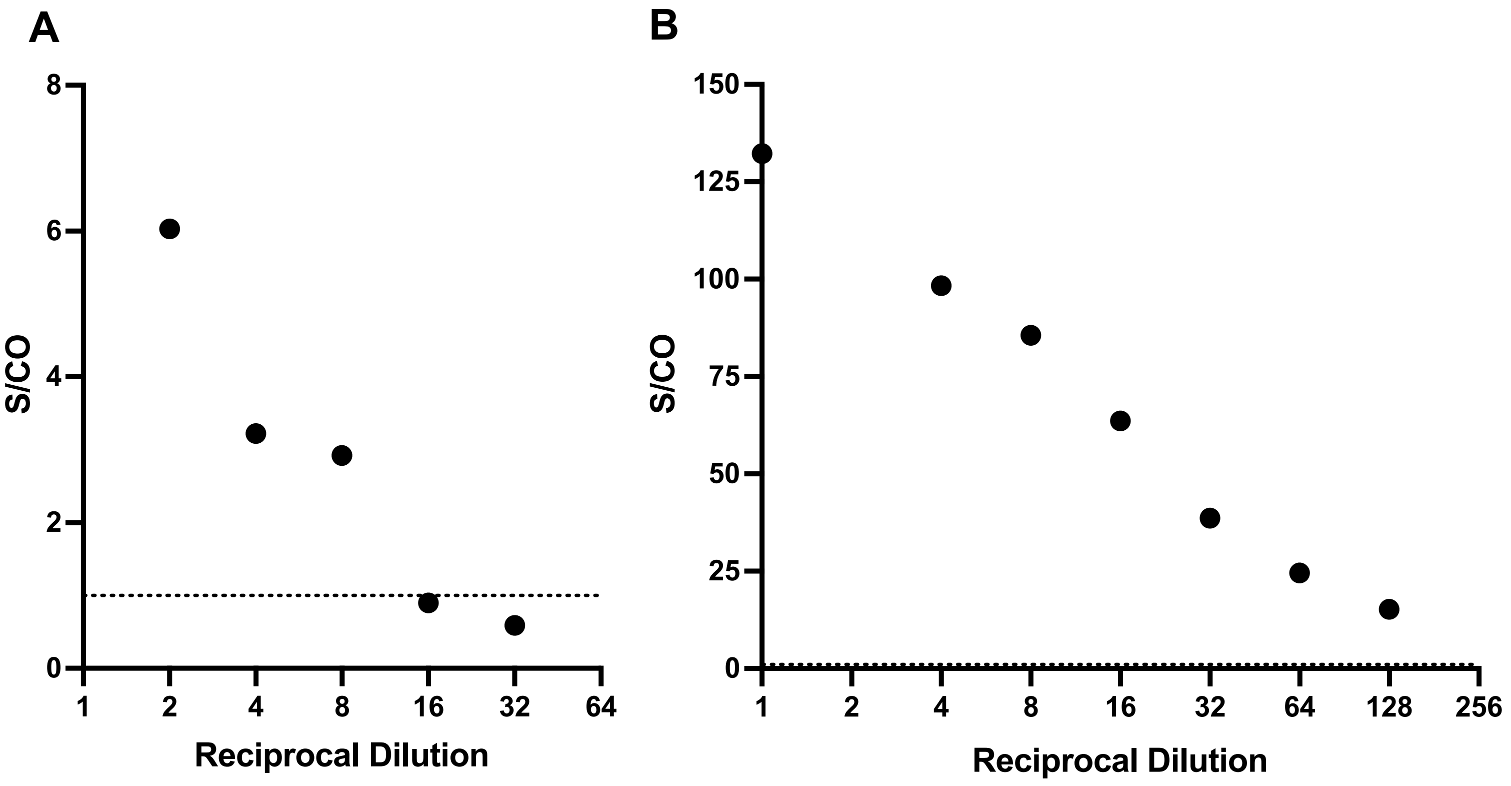

Supplemental Figure 2. Determination of S/CO values of diluted samples. (A) Samples previously testing (A) low and (B) high HTLV-1/2 CMIA positive were serially diluted with HTLV-1/2 CMIA negative sera and then S/CO values were determined by CMIA for each dilution.

Supplemental Table 1
Demographic data of re-recruited HTLV-1 positive women

Characteristic	HTLV Positive Mother of HTLV Positive 21 Month Old Child	HTLV Positive Mother of HTLV Positive 18 Month Old Child	HTLV Positive Mother of HTLV Negative 19 Month Old Child	HTLV Positive Mother of HTLV Negative 14 Month Old Child
Age	24	25	29	30
Parity	0	1	1	0
Chronic Illnesses	None	None	None	None
Delivery	Spontaneous	Unknown	Spontaneous	Spontaneous
Medication Exposure in Pregnancy	Antibiotics (delivery)	Unknown	None	Antibiotics (delivery)
Previous Miscarriage	None	None	None	1
Breastfeeding at Birth	Yes	Yes	Yes	Yes
Feeding at 6 Weeks	Mixed	Unknown	Breastmilk Only	Mixed
Education	Tertiary	Secondary	Tertiary	Tertiary

Supplemental Table 2
Cost analysis of quarterly pooled HTLV testing using different cut-off values

Reagent	Reagent cost	Cut-off ≥1 S/CO		Cut-off ≥4 S/CO		Cut-off ≥7 S/CO	
		Reagent quantity required[1]	Reagent cost	Reagent quantity required[2]	Reagent cost	Reagent quantity required[3]	Reagent cost
Pre-trigger Solution	$128.87	1	$128.87	1	$128.87	1	$128.87
Trigger Solution	$112.64	1	$112.64	1	$112.64	1	$112.64
Wash Buffer	$50.87	1	$50.87	1	$50.87	1	$50.87
Probe Conditioner	$85.86	1	$85.86	1	$85.86	1	$85.86
Reaction Vessel	$0.09	354	$31.86	324	$29.16	274	$24.66
Sample Cup	$0.05	354	$17.70	324	$16.20	274	$13.70
*r*HTLV-I/II reagent test	$3.43	354	$1,214.22	324	$1,111.32	274	$939.82
*r*HTLV-I/II control	$291.19	2	$582.38	2	$582.38	2	$582.38
*r*HTLV-I/II calibrator	$151.01	2	$302.02	2	$302.02	2	$302.02
Western blot (36 tests)[4]	$1,397.00	1	$1,397.00	1	$1,397.00	1	$1,397.00
Total Cost:			$3,923.42		$3,816.32		$3,637.82

[1]Pooled testing for ≥1 S/CO cut-off includes 116 pool tests, 184 de-pooled tests (from anticipated 23 HTLV-positive pools), 46 confirmatory retests (from anticipated 23 HTLV-positive samples), and 8 control tests (2 per quarter).
[2]Pooled testing for ≥4 S/CO cut-off includes 116 pool tests, 160 de-pooled tests (from anticipated 20 HTLV-positive pools), 40 confirmatory retests (from anticipated 20 HTLV-positive samples), and 8 control tests (2 per quarter).
[3]Pooled testing for ≥7 S/CO cut-off includes 116 pool tests, 120 de-pooled tests (from anticipated 15 HTLV-positive pools), 30 confirmatory retests (from anticipated 15 HTLV-positive samples), and 8 control tests (2 per quarter).
[4]The smallest Western blot kit size available is 36 tests.
